# A prototype-augmented few-shot segment anything model for field phenotyping of lettuce germplasm from UAV imagery

**DOI:** 10.1016/j.plaphe.2026.100276

**Published:** 2026-08-26

**Authors:** Tian Xia, Jiahui Qi, Songtao Ban, Mengen Yuan, Linyi Li, Dong Hu, Jifeng Ning, Guotao Huo, Hui Yang, Shuping He, Guojun Ge, Shiwei Wei, Minglu Tian

**Affiliations:** a School of Computer and Information Engineering, Institute for Artificial Intelligence, Shanghai Polytechnic University, Shanghai, 201209, China; b Institute of Agricultural Science and Technology Information, Shanghai Academy of Agricultural Sciences, Shanghai, 201403, China; c Shanghai Agrobiological Gene Center, Shanghai, 201106, China; d CAS Centre for Excellence in Molecular Plant Sciences, Shanghai Institute of Plant Physiology and Ecology, Chinese Academy of Sciences, Shanghai, 200032, China; e College of Information Engineering, Northwest A&F University, Yangling, Shaanxi, 712100, China; f Shanxi Engineering Research Center of Agricultural Information Intelligent Perception and Analysis, Yangling, Shaanxi, 712100, China; g Key Laboratory of Intelligent Agricultural Technology (Yangtze River Delta), Ministry of Agriculture and Rural Affairs, PR China, Shanghai, 201403, China

**Keywords:** Lettuce, Few-shot learning, UAV remote sensing, High-throughput phenotyping, Image segmentation

## Abstract

Accurate phenotypic acquisition determines the effectiveness of germplasm screening, while the efficiency and accuracy of image segmentation directly affect the quality of phenotypic parameter extraction. While foundation models like Segment Anything Model 2 (SAM2) have achieved remarkable success in general tasks, their performance in complex field environments is often limited by a lack of crop-specific visual priors. To achieve high-precision segmentation with minimal data, few-shot learning has gained traction as a strategic alternative to manual-intensive training; however, existing approaches still struggle with weak feature generalization and imprecise boundary delineation when facing highly heterogeneous lettuce germplasm. To address these issues, this study proposes a segmentation model that integrates Prototype Feature-Enhanced Prompting and a Complex Gated Spatial-Channel Attention Mechanism to improve lettuce image segmentation accuracy under complex conditions. Specifically, a Prototype Feature-Enhanced Prompting Module (PFEM) is designed to retrieve historical visual prototypes from an external feature library and fuse them into prompt embeddings, thereby introducing instance-level priors. In addition, a Complex Gated Spatial-Channel Attention Module (CGSC) is embedded in the decoder to enhance boundary perception and key feature awareness. Finally, a Composite Loss Function (CLF) is developed to jointly optimize pixel classification and region overlap.

Experimental results showed that, under the 1-shot evaluation setting on a self-built open-field lettuce dataset, the proposed model achieved an accuracy of 99.57%, a precision of 99.17%, a recall of 96.63%, a Dice coefficient of 97.90%, an IoU of 95.85%, and a Boundary IoU of 94.71%. Compared with the evaluated methods, these metrics were higher by 1.16–15.08, 1.16–16.03, 1.21–15.68, 1.56–16.69, 0.72–14.97, and 2.26–14.99 percentage points, respectively. Based on these high-precision segmentation results, fifteen morphological, color, and texture phenotypic parameters were automatically extracted. The proposed method provides a high-precision, low-sample-dependence solution for high-throughput plant phenotyping, offering robust technical support for germplasm screening.

## 1 Introduction

Lettuce (*Lactuca sativa* L.) has become a globally widely cultivated and economically important crop due to its crisp texture and high nutritional value^[^[1], [2], [3], [4], [5], [6]^]^. To meet growing market demands and ensure sustainable industry development, modern breeding programs are increasingly prioritizing the rapid selection of varieties with superior agronomic traits and environmental adaptability^[^[7], [8], [9]^]^. In this context, efficient and accurate phenotypic information extraction plays a pivotal role in characterizing germplasm resources and accelerating the breeding cycle of new varieties [10].

In breeding scenarios, experimental fields typically span considerable areas to accommodate diverse germplasm resources with wide-ranging phenotypic variations [11]. While traditional manual phenotyping is time-consuming and labor-intensive [12], unmanned aerial vehicle (UAV)-based remote sensing has emerged as a key technology for high-throughput phenotyping, offering efficient, large-scale, and non-destructive data acquisition [[13], [14], [15], [16]]. Crucially, the translation of these raw aerial images into actionable phenotypic data necessitates a prerequisite step: the precise segmentation of pure plant pixels from the background. However, this process is often hindered by complex field conditions—such as weed interference, background clutter, and significant variations in color, leaf shape, and structure among cultivars—which reduce segmentation accuracy and subsequently limit the reliability of phenotypic extraction. Consequently, achieving high-precision lettuce image segmentation remains a major challenge for accurate phenotyping.

As a core task in computer vision, image segmentation is essential to addressing these challenges. In breeding practice, due to complex backgrounds and inter-varietal differences, researchers typically need to construct large-scale, fine-grained annotated datasets for segmentation tasks [17,18], which demands substantial time and labor [19]. Few-shot learning (FSL) provides a pivotal solution by enabling models to generalize from a limited number of labeled samples, making it highly valuable under data-scarce conditions[[20], [21], [22], [23]]. Recent advancements in this domain have largely focused on optimizing feature representation and metric learning strategies. Representative approaches typically employ meta-learning frameworks to construct compact class prototypes, performing segmentation by measuring the similarity between pixel features and these prototypes [24]. To further reduce annotation dependency and adapt to variable targets, other studies have integrated hierarchical prior knowledge with similarity feature enhancement modules [25]. Additionally, techniques based on boundary tracking and region growing have been effectively utilized to extract fine-grained morphological traits, such as leaf shape and venation, from raw imagery [26]. Deep learning-based semantic segmentation has also been applied to abnormal-leaf segmentation in hydroponic lettuce for robotic sorting [27]. Although these approaches demonstrate the value of task-specific segmentation in agricultural applications, they do not directly address few-shot generalization under complex open-field backgrounds or the preservation of fine target boundaries.

Meanwhile, general segmentation models such as the Segment Anything Model 2 (SAM2) have demonstrated strong zero-shot generalization ability through large-scale pretraining, achieving remarkable results in open-domain scenarios [[28], [29], [30], [31]]. However, the performance of general-purpose vision foundation models can be dataset-dependent in heterogeneous agricultural fields [32]. Lacking domain-specific semantic knowledge, these models struggle to distinguish crops from visually similar weeds (the “green-on-green” problem) [33] and frequently fail to preserve fine-grained boundary details amidst environmental noise such as shadows and complex textures, thereby compromising the reliability of phenotype extraction [34].

Attention mechanisms enhance a model's ability to focus on key regions through adaptive feature reweighting, offering an effective solution to these challenges [35,36]. In the spectral domain, channel attention has been successfully employed to optimize feature weights via inter-channel interactions, effectively suppressing soil background interference [37]. Complementarily, spatial and geometric attention mechanisms have been developed to refine feature extraction, facilitating tasks ranging from accurate seed counting and distribution localization [38] to high-precision, organ-level 3D plant segmentation that fuses complex geometric relationships [39]. These studies demonstrate that attention mechanisms exhibit strong adaptability in complex agricultural scenarios. Nevertheless, how to dynamically integrate multiple types of attention to maximize their complementary benefits remains an open question.

Despite the widespread adoption of computer vision in crop phenotyping [ [40,41]], several limitations persist when deploying models in complex open-field scenes. In practical applications, such as the high-throughput phenotyping of large-scale lettuce germplasm resources, existing few-shot learning (FSL) methods struggle with three major challenges. First, extreme intra-class diversity poses a primary difficulty. The substantial variation in leaf morphology, color, and structural patterns among different cultivars makes it difficult for models trained with limited annotations to learn consistent representations, resulting in poor generalization. Second, complex background environments introduce significant interference. Biotic factors like weeds exhibit visual characteristics highly similar to lettuce, increasing misclassification risks, while abiotic components—such as withered leaves, field labels, and strong specular reflections on plastic mulch—introduce severe optical noise. Third, boundary ambiguity remains a critical issue. Occlusions among overlapping leaves, irregular edges, and non-uniform lighting collectively obscure target contours, rendering current approaches insensitive to fine structural details. Together, these factors highlight the necessity of incorporating domain-specific prior knowledge and targeted feature enhancement mechanisms into baseline segmentation architectures.

To address these limitations, this study proposes a Prototype-Augmented Few-Shot Segment Anything Model (PSAM) built upon the SAM2 framework. The proposed model incorporates three key innovations:

(1) a Prototype Feature-Enhanced Prompting Module (PFEM) that retrieves instance-level visual priors from an external feature library to enhance crop-specific discriminative capability;
(2) a Complex Gated Spatial-Channel Attention Module (CGSC) that dynamically fuses spatial and channel features to improve perception of plant boundaries and fine structural details;
(3) a Composite Loss Function (CLF) that jointly optimizes hard samples and region overlaps, enhancing segmentation robustness under class imbalance.

Under the 1-shot evaluation setting, the proposed model outperformed the evaluated methods on the self-constructed open-field lettuce dataset, providing a feasible approach for annotation-efficient plant phenotyping.

## 2 Materials and methods

### 2.1 Data acquisition and processing

#### 2.1.1 Experimental materials and data collection

In this study, a total of 918 lettuce germplasm accessions provided by the Shanghai Agrobiological Gene Center (SAGC) were selected as experimental materials. The field experiment was conducted in Jinshan District, Shanghai, China (30°48′N, 121°12′E), covering an area of approximately 1 ha. All seeds were germinated and sown on April 26, 2024, and transplanting was completed on May 25, 2024. The planting layout was established with a plant spacing of 25 cm and a row spacing of 30 cm, with a total of 1884 individual plots. Each plot contained 30 lettuce plants of the same genotype, arranged in a randomized distribution with replicated plantings. In total, 56,520 lettuce plants were cultivated, including Romaine, Butterhead, Crisphead, and Loose-leaf types, which were further categorized by leaf color (e.g., green, dark green, red, and yellow). The considerable phenotypic heterogeneity across lettuce germplasm posed significant challenges for segmentation model generalization, while also providing an ideal benchmark for performance evaluation.

To efficiently acquire high-resolution images suitable for phenotypic analysis, aerial image data collection was performed using a DJI Matrice 300 RTK unmanned aerial vehicle (UAV) equipped with a Zenmuse P1 full-frame RGB camera (DJI, Shenzhen, China). The flight parameters were set as follows: altitude = 18 m, forward overlap = 80%, and side overlap = 70%. Under this configuration, the dataset achieved a ground sampling distance (GSD) of 0.1 cm/pixel, which provided sufficient spatial resolution to capture fine-grained leaf morphological features of individual lettuce plants.

#### 2.1.2 Data preprocessing and dataset construction

**Fig. 1:**
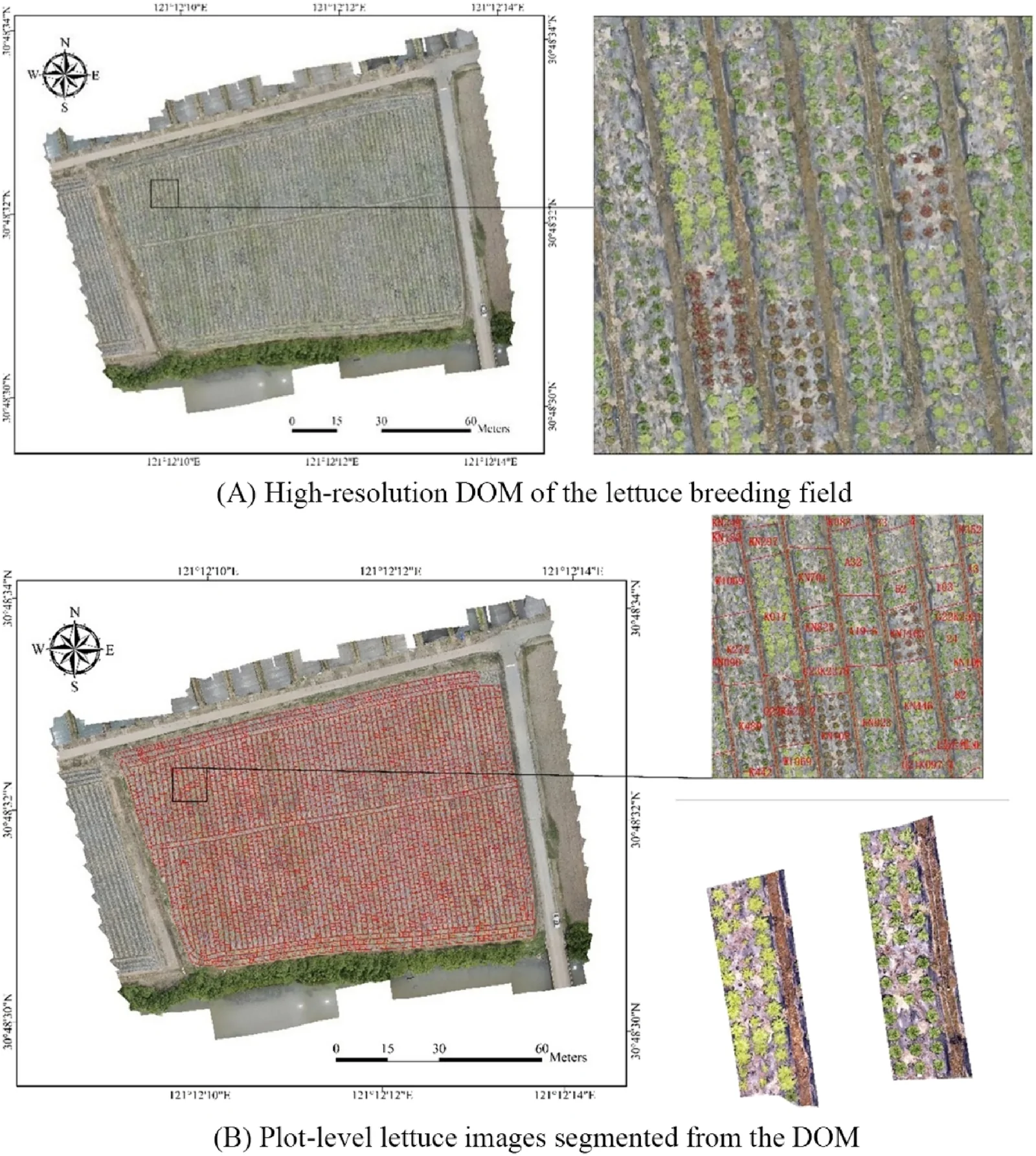
Field data acquisition and preprocessing workflow. (A) High-resolution digital orthophoto map (DOM) of the experimental lettuce field. (B) Individual plot images generated after boundary delineation and segmentation, used for single-plant phenotyping.

A high-resolution Digital Orthophoto Map (DOM) covering the entire experimental field was generated using Pix4Dmapper software (Version 4.5, Pix4D S.A., Prilly, Switzerland) (Fig. 1(A)). To minimize imaging bias caused by acquisition time and lighting variations, the DOM was preprocessed using Contrast-Limited Adaptive Histogram Equalization (CLAHE) and a white balance correction algorithm, both implemented in Python using the OpenCV library. Field plot boundaries were first manually delineated and subsequently employed to segment the DOM using ArcGIS (Version 10.8, Esri, Redlands, CA, USA), generating a total of 1884 individual plot images, as illustrated in Fig. 1(B), providing standardized data for subsequent single-plant phenotyping.

**Fig. 2:**
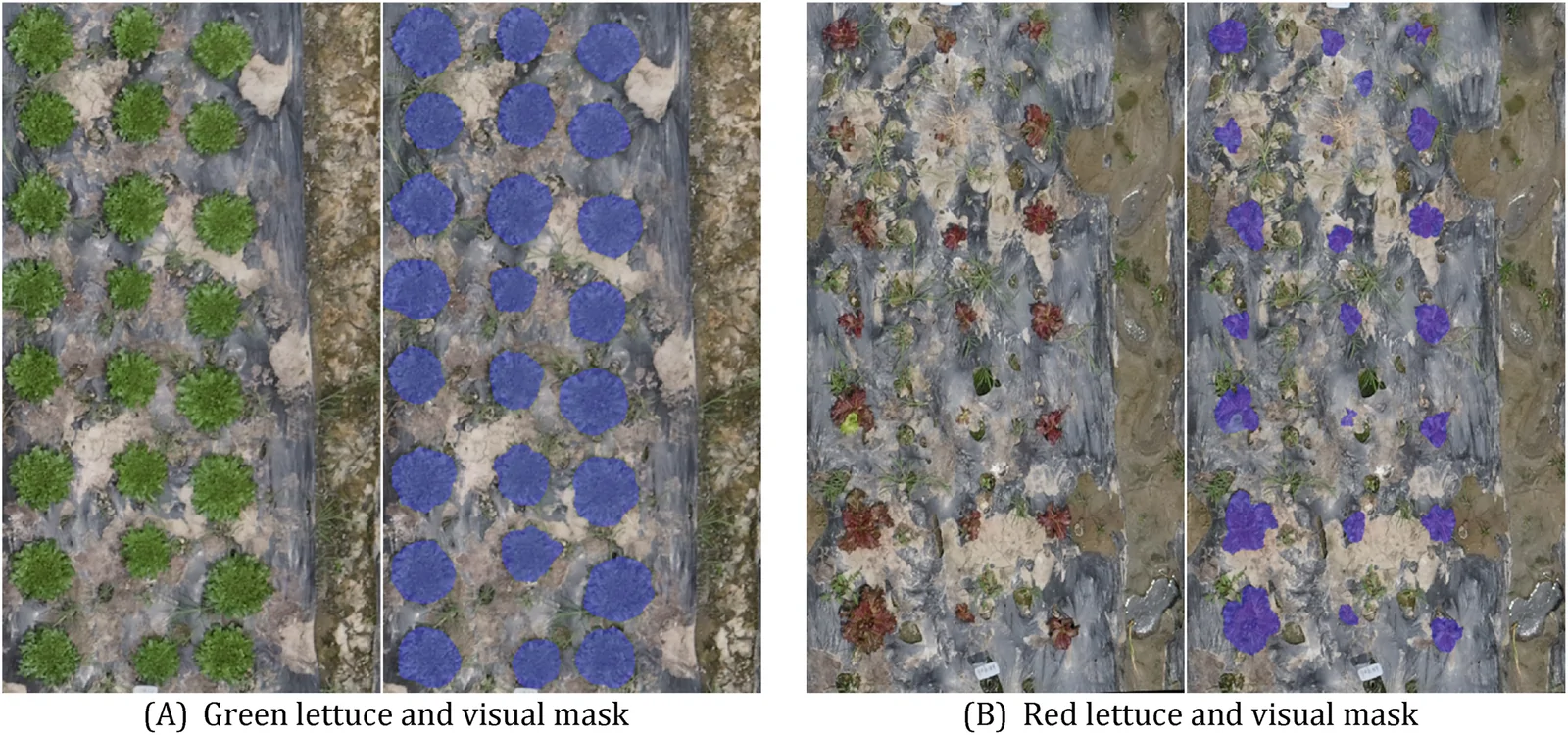
Representative examples of plot images and their annotated segmentation masks for model training and evaluation. The left panel displays the original RGB image under complex field conditions. The right panel presents the corresponding mask visualization, where the binary segmentation result is overlaid on the original image, depicting the lettuce plant region with a translucent blue mask and a clear boundary contour. (A) The original image of green lettuce and the corresponding manually refined mask. (B) The original image of red lettuce and the corresponding manually refined mask.

A two-stage procedure was adopted for pixel-level annotation. First, the Segment Anything Model 2 (SAM2) was used to automatically generate initial lettuce masks. Then, annotators refined these results manually using LabelMe, ensuring accurate delineation between lettuce plants and background elements such as soil, plastic mulch, shadows, and weeds. This process produced high-quality binary mask labels. Representative examples of plot images and their corresponding overlay visualizations of segmentation masks are shown in Fig. 2.

For few-shot segmentation tasks, dataset partitioning was conducted in consultation with breeding experts. Considering variations in leaf color (light green, dark green, deep red, yellow, etc.), lettuce type (Romaine, Crisphead, Loose-leaf, etc.), and spatial distribution patterns, a systematic split was performed. From the total 1884 plot images, 65 images with diverse visual characteristics and fine annotations were selected as the training set to construct the model's core prior knowledge. Another 325 images were randomly selected as the validation set for hyperparameter tuning and model selection. The remaining 1494 images constituted the test set for unbiased performance evaluation. Notably, the external feature library for prototype retrieval was constructed solely from training set instances. This design mimics the breeding scenarios, where only a small number of labeled samples are available for rapid model adaptation to new varieties and environments, thereby rigorously assessing model generalization.

### 2.2 Prototype-augmented few-shot segment anything model

To address the challenge of lettuce image segmentation under complex field backgrounds, we propose the Prototype-Augmented Few-Shot Segment Anything Model (PSAM). SAM2 struggles to distinguish foreground and background accurately due to the lack of instance-level priors for specific lettuce cultivars, which often results in blurred and incomplete segmentation boundaries.

**Fig. 3:**
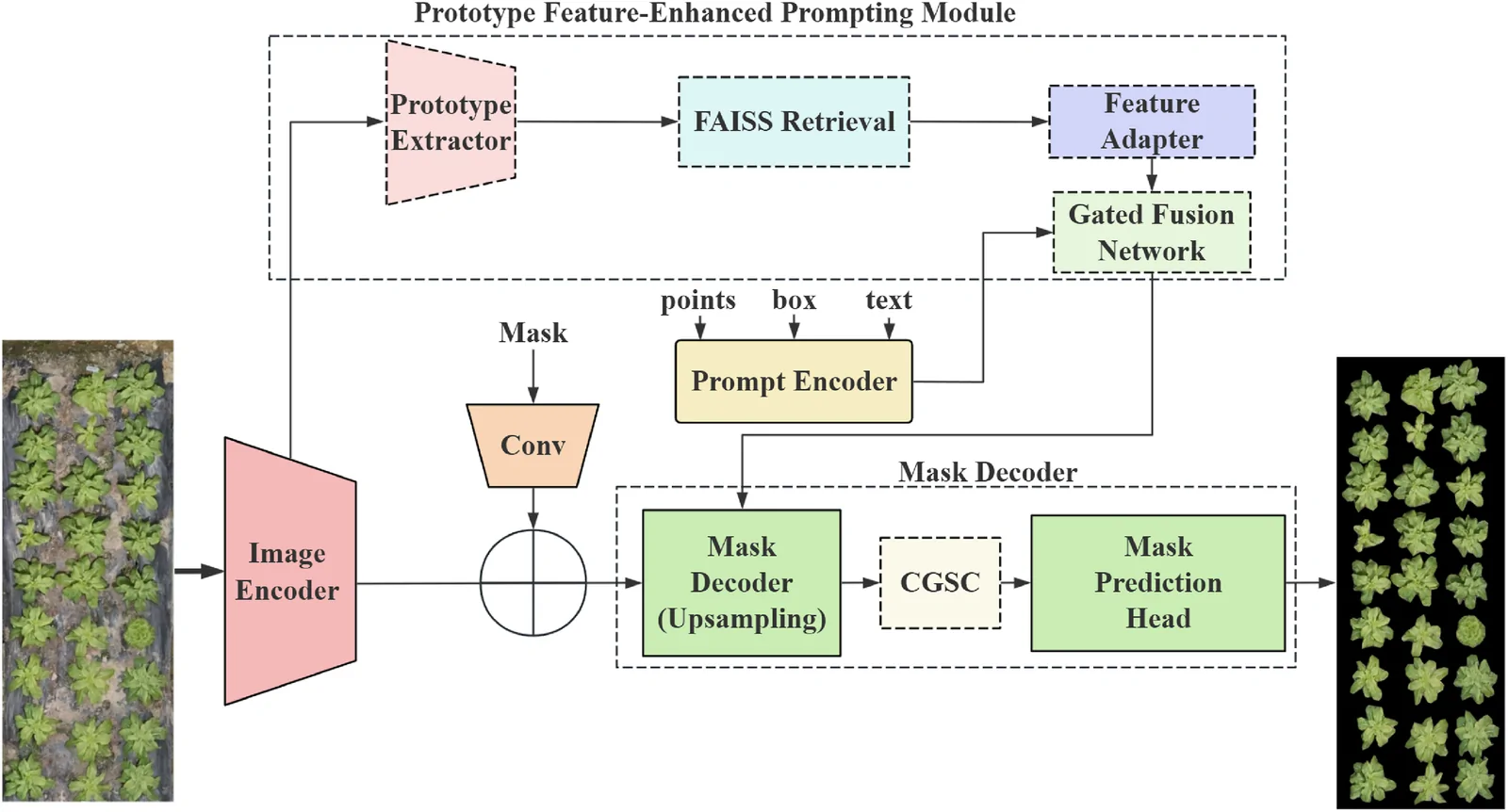
Overall architecture of the proposed Prototype-Augmented Few-Shot Segment Anything Model (PSAM). The model follows an encoder-prompt-decoder design, integrating a Prototype Feature-Enhanced Prompting Module (PFEM) for prior knowledge injection and a Complex Gated Spatial-Channel Attention Module (CGSC) for boundary-aware feature refinement. Dashed boxes indicate the novel modules introduced in PSAM, while solid boxes represent the unchanged SAM2 core components.

To overcome the core challenges of extreme intra-class diversity, high background similarity, and complex edge conditions, PSAM integrates two complementary innovations: a Prototype Feature-Enhanced Prompting Module (PFEM) and a Complex Gated Spatial-Channel Attention Module (CGSC). As shown in Fig. 3, PSAM follows an encoder-prompt-decoder design to maintain compatibility with SAM2 pretrained weights. Specifically, the image encoder extracts visual features. The prototype prompts generated by PFEM are first projected to match SAM2's native embedding dimension ($d_{model}=256$). They are then dynamically fused with the user's sparse prompt embeddings—which have inherently undergone SAM2's standard positional encoding—via a Gated Fusion Network within the prompt encoder. Subsequently, the CGSC module is inserted after the two-stage upsampling layers in the mask decoder, prior to the final mask prediction heads, to refine multi-scale features. This design integrates domain-specific priors while preserving the original core structure and pre-trained weights of SAM2. PSAM introduces an external prototype knowledge mechanism and an internal feature refinement process. PFEM retrieves historical prototype features from an external feature library to inject instance-level priors, while CGSC enhances feature representations within the mask decoder through dual spatial-channel attention. Together, these modules allow PSAM to achieve high-precision segmentation of field-grown lettuce with only a few annotated samples.

#### 2.2.1 Prototype Feature-Enhanced Prompting Module

**Fig. 4:**
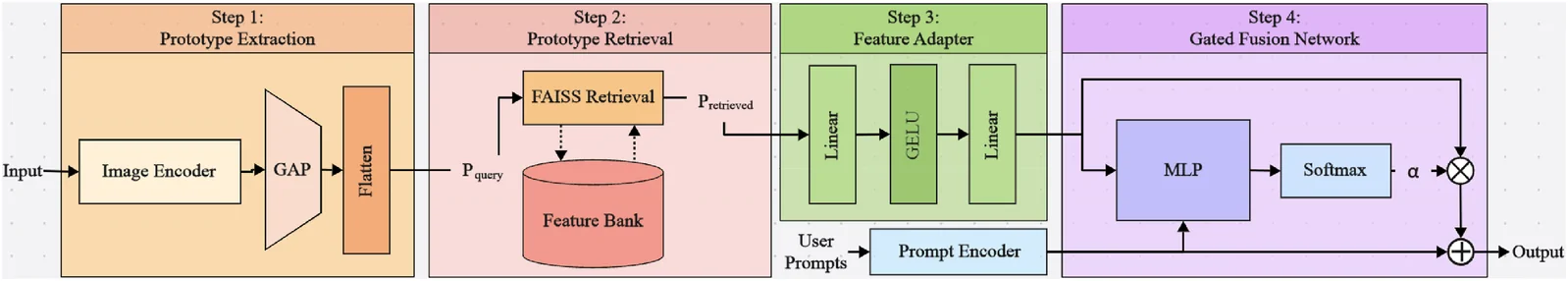
Structure of the Prototype Feature-Enhanced Prompting Module (PFEM). The module operates through four sequential steps: instance prototype feature extraction, efficient prototype retrieval via FAISS, feature dimension adaptation, and attention-guided prompt fusion with user inputs.

Lettuce germplasm exhibits rich phenotypic diversity. To overcome segmentation performance limitations caused by large intra-class variation among different cultivars, this study designs a Prototype Feature-Enhanced Prompting Module (PFEM). PFEM injects data-driven instance-level priors via an external feature library, enhancing the model's adaptability to specific agricultural scenarios. As illustrated in Fig. 4, PFEM operates through four tightly connected steps: instance prototype feature extraction, efficient prototype retrieval, feature adaptation, and attention-guided prompt fusion.

Extracting discriminative instance representations from images is critical for effective prototype feature augmentation. To this end, deep feature maps $F_{\text{img}}\in R^{C\times h\times w}$ output by the image encoder are utilized. While these feature maps contain rich spatial details, their high dimensionality hinders direct similarity comparisons. A projection function P is therefore designed, applying global adaptive average pooling followed by flattening to convert $F_{\text{img}}$ into a fixed-dimension prototype feature vector $p\in R^{D}$:

(1) $$\begin{array}{c} p=P\left(F_{\text{img}}\right)=Flatten\left(GAP\left(F_{\text{img}}\right)\right) \end{array}$$

This operation preserves core feature responses while embedding each image instance in a high-dimensional semantic space, providing a foundation for subsequent retrieval. All extraction operations are executed in no-gradient mode to maintain backbone stability.

**Fig. 5:**
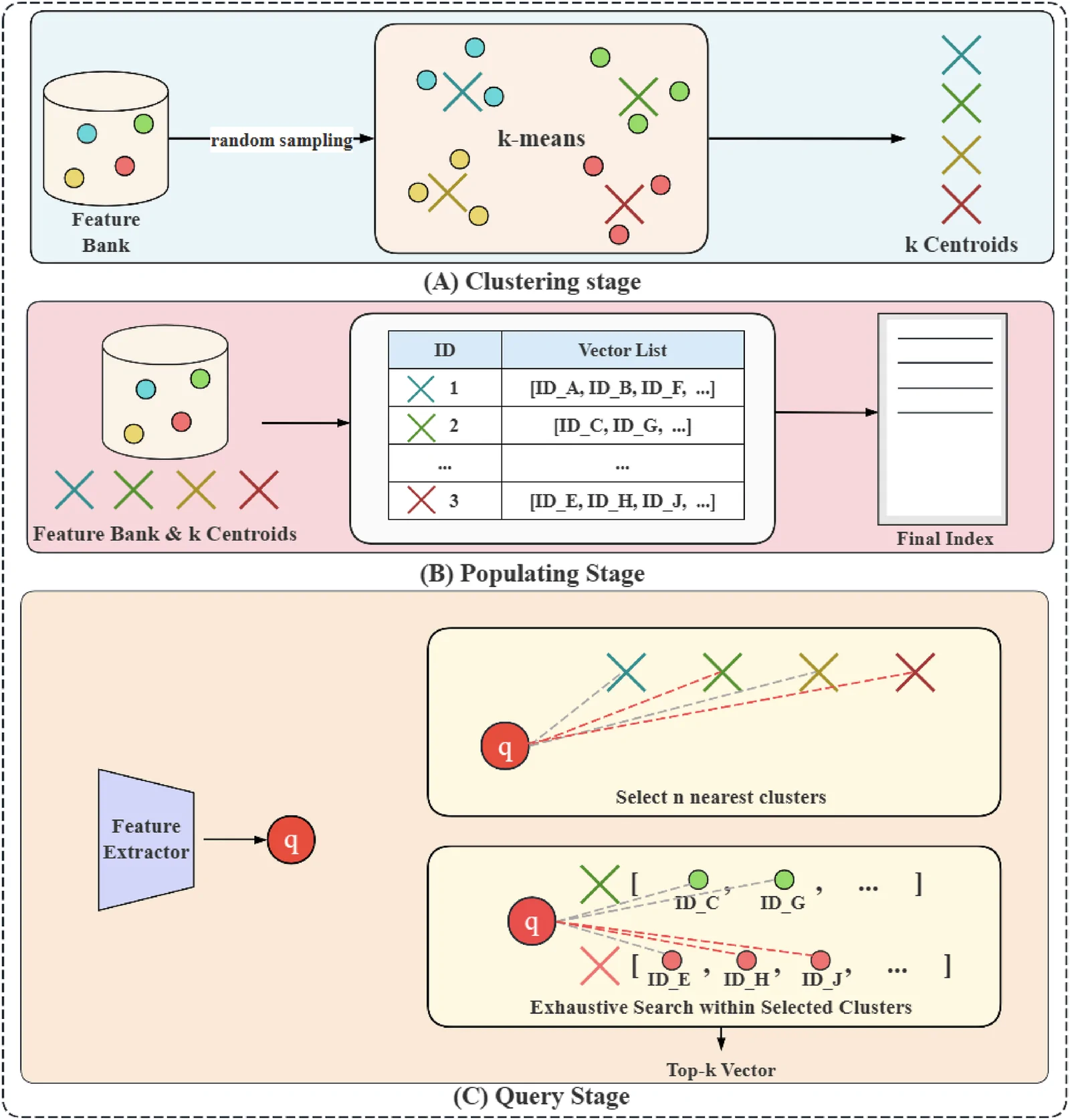
Workflow of the prototype feature retrieval process using the FAISS library. (A) Feature space partitioning via k-means clustering. (B) Construction of an inverted index for efficient searching. (C) Query within the nearest clusters to retrieve the most relevant visual prototype.

To improve computational efficiency, an approximate nearest neighbor search is implemented using the FAISS library [42] for dense vector similarity search. The external prototype feature library employed a dynamic construction and update mechanism. To ensure strict dataset separation, it was constructed and updated exclusively using the 65 annotated training images, whereas the 1-shot and 5-shot settings refer to the number of annotated support images provided in each evaluation episode. When the model's prediction confidence exceeds a predefined threshold (e.g., object score logits > 0.5) during training, the newly extracted 1D prototype from the current image is appended to the library. To manage memory and maintain feature relevance, a frequency-based tracking mechanism (access count) acts as a least frequently used (LFU)-like policy, evicting the least frequently retrieved prototypes upon reaching a predefined maximum capacity (set to 1000 in our implementation). Furthermore, the FAISS inverted indices are dynamically rebuilt at the end of each training epoch to ensure the retrieval space remains up-to-date. Crucially, to prevent any potential data leakage and ensure a rigorous evaluation, the feature library is strictly frozen during the testing phase. No representations from the test set are added to the library, ensuring the model relies solely on the priors learned from the limited training samples. The workflow (Fig. 5) first partitions the feature space using k-means clustering (Fig. 5(A)), then constructs an inverted index (Fig. 5(B)), and finally searches only a limited set of nearest clusters during query (Fig. 5(C)). The search is constrained to a subset $C_{\left(q,n_{probe}\right)}$, optimizing the retrieval target as:

(2) $$\begin{array}{c} p^{*}=\arg\min_{p_{i}\in C_{\left(q,n_{probe}\right)}}{\left\|q-p_{i}\right\|}_{2}^{2} \end{array}$$

where $p^{*}\in R^{D}$ is the retrieved optimal prototype vector, $p_{i}$ is any vector in the subset $C_{\left(q,n_{probe}\right)}$ of the nearest $n_{probe}$ clusters, and $q\in R^{D}$ is the query vector generated from the input image $I_{q}$. The L2 norm ${\left\|\cdot \right\|}_{2}^{2}$ computes the squared Euclidean distance, and $\arg\min_{p_{i}\in C_{\left(q,n_{probe}\right)}}\left(\cdot \right)$ selects the vector minimizing this distance. In this study, the number of clusters $k_{clusters}$ = 8 and query clusters $n_{probe}$ = 4, providing a practical balance between prototype diversity and retrieval efficiency, with indices rebuilt every training epoch to ensure up-to-date retrieval.

Since the retrieved prototype feature $p^{*}\in R^{D}$ (D = 4096) does not match the decoder embedding dimension ($d_{\text{model}}$ = 256), a lightweight feature adapter $A:R^{D}\to R^{d_{\text{model}}}$ is designed. This small MLP with two hidden layers and GELU activation maps the generalized prototype nonlinearly to a task-specific prompt embedding:

(3) $$\begin{array}{c} P_{\text{adapted}}=A\left(p^{*}\right)=W_{2}\cdot \text{GELU}\left(W_{1}p^{*}+b_{1}\right)+b_{2} \end{array}$$

where $W_{1}$, $b_{1}$, $W_{2}$, $b_{2}$ are learnable parameters.

Finally, the adapted prototype prompt $P_{adapted}\in R^{d_{model}}$ is dynamically fused with sparse prompt embeddings ${Ε}_{sparse}\in R^{N_{p}\times d_{model}}$ via an attention mechanism. Specifically, this mechanism is implemented using a lightweight Gated Fusion Network (comprising an MLP and a Softmax layer). The network processes the feature representations to compute correlations, and the Softmax layer normalizes these values into a dynamic attention weight α∈(0,1). The fusion process is formulated as:

(4) $$\begin{array}{c} P_{fused}=P_{sparse}⊕\left(\alpha ⊗P_{adapted}\right) \end{array}$$

where $P_{sparse}$ represents the embedding of the user prompt, and α denotes the learned attention weight generated by the Softmax layer.

This enables the model to balance user-provided prompts and prior knowledge learned from samples. When encountering new cultivars, PFEM leverages stored visual memory to guide segmentation decisions, substantially improving generalization for highly heterogeneous lettuce germplasm.

#### 2.2.2 Complex Gated Spatial-Channel Attention Module

**Fig. 6:**
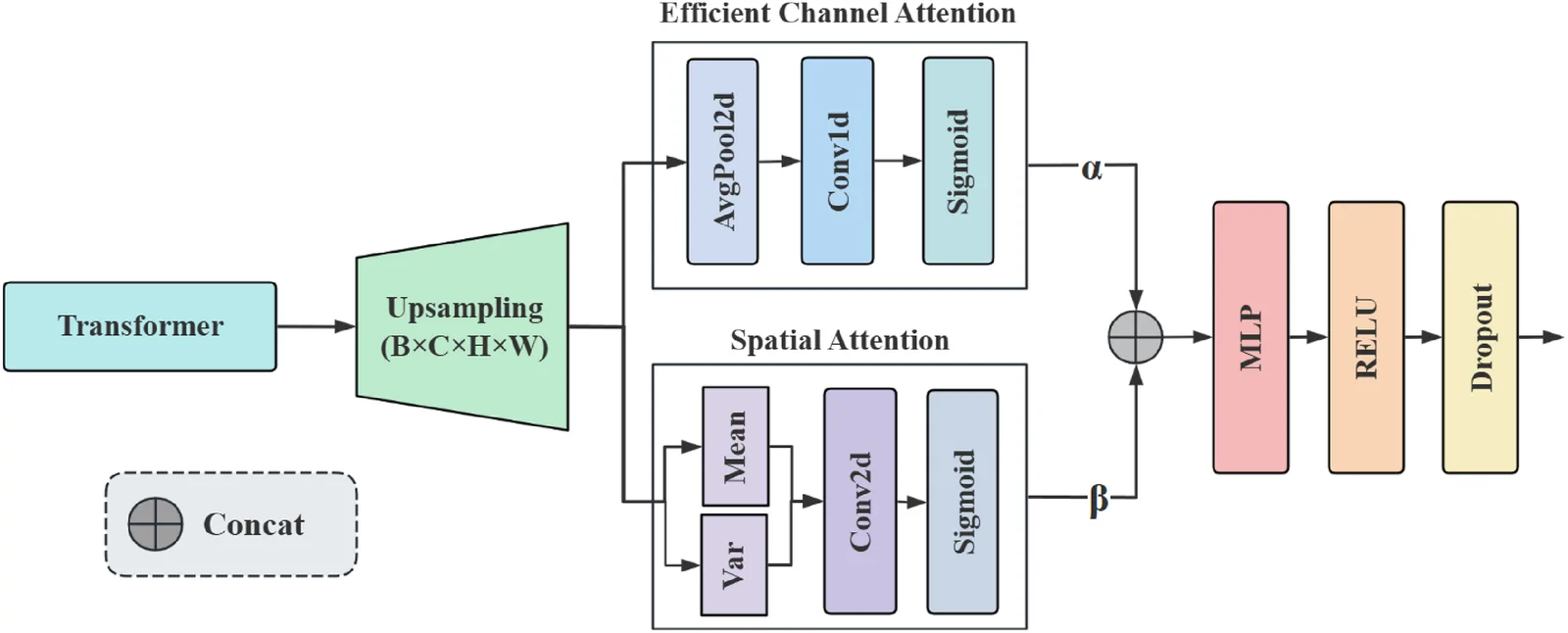
Detailed architecture of the Complex Gated Spatial-Channel Attention Module (CGSC). The module consists of a channel attention branch (modeling inter-channel dependencies), a spatial attention branch (highlighting target regions via feature statistics), and a dynamic gated fusion network to adaptively combine both attention outputs.

Field backgrounds often contain abundant interfering objects and uneven illumination (e.g., shadows), which cause feature distortion and boundary blurring. To address these challenges, this study proposes the Complex Gated Spatial-Channel Attention (CGSC) module, which dynamically integrates channel and spatial attention to enhance the decoder's perception of multi-scale, multi-dimensional features. The CGSC architecture is illustrated in Fig. 6.

The CGSC module consists of three core components designed to decouple and refine feature representations. In the channel attention branch, local cross-channel interactions are modeled via 1D convolutions. Because different feature channels encode distinct semantic patterns (e.g., structural texture versus color intensity), this branch learns to assign lower weights to channels dominated by illumination variations or shadow artifacts. This mechanism suppresses shadow interference at the feature level without reducing the spatial resolution. In the spatial attention branch, a feature-statistics-based spatial attention strategy is proposed. Specifically, the mean and variance across channels are computed at each spatial location of the input feature map. Mechanistically, the mean maps capture overall object activation to filter out background noise, while the variance maps specifically encode local feature fluctuations to locate structural edges. These computed statistical maps are then passed through a 7×7 convolution and a Sigmoid activation to produce a spatial attention weight map. Element-wise multiplication is applied to enhance the feature map, adaptively sharpening blurred leaf boundaries and highlighting target plant regions:

(5) $$\begin{array}{c} w_{s}=\sigma \left({Conv}_{7\times 7}\left(\left[\mu ;s^{2}\right]\right)\right) \end{array}$$

(6) $$\begin{array}{c} F_{ssa}=X⨀w_{s} \end{array}$$

where σ denotes the Sigmoid function, [·;·] represents concatenation, and ⨀ indicates element-wise multiplication. μ and $s^{2}$ denote the channel-wise mean and variance feature maps derived from the input X, respectively.

To adaptively fuse the two attention mechanisms, a dynamic gated fusion network is introduced. The network first extracts global context vectors for both channel and spatial attention via global average pooling, then feeds them into a small MLP followed by a Softmax function to generate normalized fusion weights [α,β]:

(7) $$\begin{array}{c} \left[\alpha ,\beta \right]=Softmax\left(G\left(\left[GAP\left(F_{eca}\right);GAP\left(F_{ssa}\right)\right]\right)\right) \end{array}$$

This gating mechanism enables the model to dynamically shift its feature refinement focus: prioritizing channel attention for interior leaf regions affected by shadows, and emphasizing spatial attention for regions requiring edge refinement.

Finally, the enhanced features are obtained by weighted fusion of the two attention outputs, combined with a residual connection and batch normalization to stabilize training:

(8) $$\begin{array}{c} X_{enhanced}=\text{BN}\left(X+\left(\alpha \cdot F_{eca}+\beta \cdot F_{ssa}\right)\right) \end{array}$$

The CGSC module is embedded after the upsampling stage in the mask decoder but before mask prediction. This ensures that features enhanced through both channel and spatial attention directly influence pixel-level classification, effectively improving segmentation accuracy and boundary consistency in complex field scenarios.

#### 2.2.3 Composite Loss Function

In lettuce image segmentation tasks under complex field conditions, model training faces two typical challenges: severe pixel imbalance between foreground plants and background, and the prevalence of hard-to-classify pixels. To address these issues, this study employs a Composite Loss Function (CLF, $L_{CLF}$) that jointly optimizes pixel-level classification and region-level overlap by combining Focal Loss [43] ($L_{Focal}$) and Dice Loss [44] ($L_{Dice}$).

Focal Loss dynamically reduces the weight of easily classified samples through a modulation factor, forcing the network to concentrate on hard-to-classify samples during training. Its formulation is defined as:

(9) $$\begin{array}{c} L_{Focal}\left(p_{t}\right)=-\alpha_{t}{\left(1-p_{t}\right)}^{\gamma }\;\log\left(p_{t}\right)\; \end{array}$$

where γ = 2 and $\alpha_{t}$ = 1. In open-field crop segmentation, pixels within the interior regions of leaves or clear backgrounds typically yield high-confidence predictions (easy samples). In this dataset, many hard-to-classify pixels occur near complex leaf margins, overlapping regions, and shadowed edges. Therefore, Focal Loss may indirectly emphasize these uncertain pixels by reducing the contribution of easy interior and background pixels. However, Focal Loss is not an explicit boundary loss and does not directly model geometric contours.

The Dice Loss component directly optimizes the overlap between predicted masks and ground truth, effectively mitigating class imbalance and promoting spatially continuous segmentation results:

(10) $$\begin{array}{c} L_{Dice}=1-\frac{2\sum_{i=1}^{N}p_{i}g_{i}}{\sum_{i=1}^{N}p_{i}^{2}+\sum_{i=1}^{N}g_{i}^{2}+\epsilon } \end{array}$$

where $p_{i}$ and $g_{i}$ represent the predicted probability and the ground truth label for the i-th pixel, respectively, N is the total number of pixels, and ϵ is a smoothing term to prevent division by zero.

Finally, the total loss is a weighted combination of the two:

(11) $$\begin{array}{c} L_{CLF}=\lambda_{Focal}L_{Focal}+\lambda_{Dice}L_{Dice} \end{array}$$

By setting $\lambda_{Focal}$ = 20 and $\lambda_{Dice}$ = 1, the contributions of each component to gradient updates are balanced, allowing the model to remain sensitive to hard-to-classify pixels while maintaining overall structural integrity.

### 2.3 Evaluation metrics for lettuce segmentation model

To comprehensively assess the performance of the model in lettuce segmentation tasks, this study adopts six widely recognized metrics, including Accuracy, Precision, Recall, Dice coefficient, Intersection over Union (IoU), and Boundary IoU. Each pixel is categorized as one of True Positive (TP), True Negative (TN), False Positive (FP), or False Negative (FN).

Accuracy is the most intuitive metric, reflecting the proportion of correctly classified pixels among all pixels, defined as:

(12) $$\begin{array}{c} \text{Accuracy}=\frac{\text{TP}+\text{TN}}{\text{TP}+\text{TN}+\text{FP}+\text{FN}} \end{array}$$

Precision measures the proportion of correctly predicted positive samples among all predicted positives:

(13) $$\begin{array}{c} \text{Precision}=\frac{\text{TP}}{\text{TP}+\text{FP}} \end{array}$$

Recall evaluates the model's ability to identify all actual positive samples:

(14) $$\begin{array}{c} \text{Recall}=\frac{\text{TP}}{\text{TP}+\text{FN}} \end{array}$$

The Dice coefficient is the harmonic mean of precision and recall, balancing both aspects:

(15) $$\begin{array}{c} \text{Dice}=2\frac{\text{Precision}\cdot \text{Recall}}{\text{Precision}+\text{Recall}} \end{array}$$

Intersection over Union (IoU) directly measures the overlap between the predicted segmentation region and the ground truth:

(16) $$\begin{array}{c} \text{IoU}=\frac{\text{TP}}{\text{TP}+\text{FP}+\text{FN}} \end{array}$$

To more accurately evaluate the model's performance on fine structural details and complex leaf margins, Boundary Intersection over Union [45] (Boundary IoU) is introduced as a supplementary metric. Unlike standard mask IoU, which is dominated by interior pixels, Boundary IoU strictly evaluates the segmentation quality within a specific distance from the contours. It is defined as:

(17) $$\begin{array}{c} Boundary\_IoU=\frac{\left|\left(G_{d}\cap P_{d}\right)\right|}{\left|\left(G_{d}\cup P_{d}\right)\right|} \end{array}$$

where $G_{d}$ and $P_{d}$ represent the sets of pixels within a specified pixel distance d from the contours of the ground truth mask G and the prediction mask P, respectively. In our experiments, the pixel distance d is dynamically set based on the image diagonal to ensure consistent evaluation across different scales.

By jointly evaluating these six metrics, the model's performance in segmenting lettuce plants under complex field conditions can be comprehensively quantified from multiple perspectives.

## 3 Results

### 3.1 Experimental settings

All experiments in this study were conducted within a server-based deep learning environment. The hardware configuration included an Intel(R) Core(TM) i5-13490F CPU @ 2.50 GHz with 32 GB RAM, and an NVIDIA GeForce RTX 4060 Ti GPU with 16 GB video memory. The software environment consisted of Windows 11 as the operating system, PyCharm as the development platform, Python 3.12 as the programming language, and PyTorch and TensorFlow as the deep learning frameworks. The specific training hyperparameters are listed in Table 1.

**Table 1:** Model training hyperparameters and optimization settings.

Train Epochs	Batch Size	Optimizer	Learning Rate(Backbone)	Learning Rate(Other)	Schedule	Dropout Rate
80	2	AdamW	3e-6	5e-6	Cosine Annealing	0.1

### 3.2 Comparison of different models for lettuce segmentation

To systematically verify the segmentation performance and generalization capability of the PSAM model under few-shot conditions, experiments were conducted under 1-shot and 5-shot settings, comparing it with current mainstream methods. In this study, 1-shot and 5-shot refer to the use of one and five annotated support images, respectively, in each evaluation episode; they do not refer to the total number of images used to construct the training-derived prototype library. These two specific settings were chosen to comprehensively evaluate the model's performance under data constraints typical in agricultural phenotyping. The 1-shot setting represents an extreme condition of data scarcity, rigorously testing the model's adaptation ability and robustness when only one annotated support image is available. Conversely, the 5-shot setting simulates a highly practical field application scenario where breeders can afford to annotate a minimal but representative batch of samples to achieve stable, high-throughput segmentation for a specific cultivar. The comparison included two categories of models: the first comprised recently proposed few-shot semantic segmentation models, such as PANet [46], CANet [17], PFENet [47], HSNet [48], HDMNet [49], Apseg [50], and DiffewS [51]; the second consisted of baseline general segmentation models represented by the official SAM2 (Hiera-B+). All models were trained and evaluated on the custom UAV-based lettuce dataset using the same training strategies and evaluation metrics to ensure a fair comparison. Table 2 presents the performance of each model across multiple evaluation metrics.

**Table 2:** Performance comparison of PSAM against state-of-the-art few-shot and general segmentation models on the custom lettuce dataset.

Methods	1-shot						5-shot					
	Acc	Prec	Recall	Dice	IoU	Boundary IoU	Acc	Prec	Recall	Dice	IoU	Boundary IoU
HSNet	86.22	85.12	82.09	83.69	81.85	80.63	87.31	86.02	83.12	84.59	82.34	81.54
PANet	84.49	83.14	80.95	81.21	80.88	79.72	85.21	84.39	81.43	82.03	81.06	80.28
CANet	86.72	85.21	82.84	84.17	83.73	82.39	87.34	85.79	83.91	84.98	84.56	83.49
PFENet	89.04	88.62	85.36	86.94	85.97	82.69	90.12	89.07	86.38	88.06	87.22	85.91
HDMNet	91.53	90.85	87.31	88.08	87.24	85.88	91.74	90.41	87.24	89.00	86.47	84.35
Apseg	91.98	91.26	89.42	90.31	89.02	87.19	92.69	92.27	90.35	91.25	89.98	88.20
DiffewS	98.41	98.01	95.42	96.34	95.13	92.45	99.31	99.12	97.69	98.21	96.81	94.66
SAM2	94.83	94.14	89.94	92.80	90.98	89.24	95.18	95.26	90.99	94.79	92.42	90.17
PSAM	**99.57**	**99.17**	**96.63**	**97.90**	**95.85**	**94.71**	**99.64**	**99.23**	**97.90**	**98.28**	**96.92**	**95.84**

*Note:***The best results are highlighted in bold.**

The experimental results demonstrate that PSAM achieves superior performance under both 1-shot and 5-shot settings. Specifically, in the 1-shot scenario, the Dice score reaches 97.90%, surpassing the strongest baseline, DiffewS, by 1.56% and the original SAM2 by 5.10%. This performance gain can be attributed to two complementary factors. First, the prototype feature library introduces crop-specific instance priors into SAM2, alleviating its lack of domain-specific knowledge under few-shot conditions. Second, CGSC enhances boundary-sensitive feature representations in the decoder, leading to more accurate separation of adjacent plants and suppression of background interference.

**Fig. 7:**
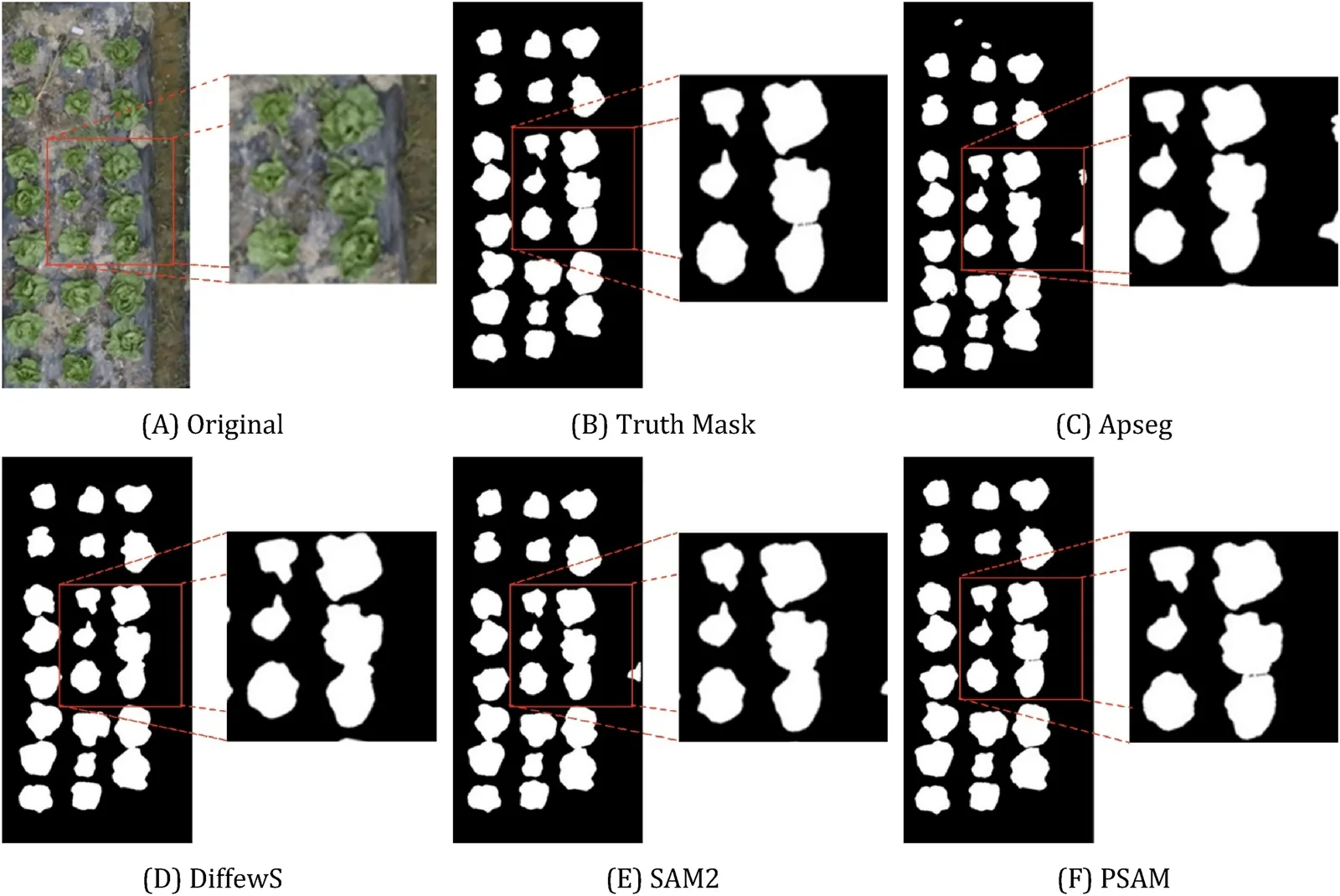
Visual comparison of segmentation results from different models under challenging field conditions. (A) Original image. (B) Ground truth mask. (C) Prediction by Apseg. (D) Prediction by DiffewS. (E) Prediction by SAM2. (F) Prediction by the proposed PSAM. For each panel, a zoomed-in view of the complex leaf margins (highlighted by the red box) is provided on the right to explicitly demonstrate the models' capabilities in boundary refinement. As shown in the insets, PSAM exhibits superior boundary delineation and effectively suppresses background interference compared to the baselines.

To provide a more intuitive demonstration of PSAM's performance advantage, several challenging segmentation cases were selected, as shown in Fig. 7.

Fig. 7 presents qualitative comparisons of different models under complex field conditions. Specifically, to highlight the models' performance on highly complex and heterogeneous leaf margins, we provide zoomed-in views of the boundary regions (indicated by red boxes). As clearly observed in the insets, Apseg suffers from noticeable false positives and under-segmentation between adjacent plants. DiffewS reduces background misclassification but still exhibits boundary merging and edge blurring in densely planted regions. The SAM2 baseline shows both background interference and incomplete separation of neighboring plants, highlighting the limitations of general-purpose models in agricultural scenes. In contrast, PSAM effectively suppresses background-induced false positives and recovers the highly irregular leaf contours. The clear separation of adjacent plants shown in the zoomed-in views demonstrates the superior boundary delineation in challenging field environments. These results validate the effectiveness of the proposed method: the prototype feature enhancement module significantly improves feature discrimination by introducing domain-specific visual priors, and the composite attention mechanism enhances boundary detail perception, improving segmentation of lettuce edges. Overall, PSAM not only performs excellently in quantitative metrics but also substantially improves segmentation quality in complex agricultural environments.

### 3.3 Ablation study

Table 3 presents the ablation results of key components in PSAM. Introducing each module individually leads to consistent performance improvements over the SAM2 baseline, indicating their independent effectiveness. Specifically, the PFEM yields the largest single-module gain, highlighting the importance of instance-level priors under few-shot conditions. The CGSC module further improves boundary sensitivity, while the CLF provides additional gains by emphasizing hard-to-classify pixels. Specifically, the CLF-only configuration increased Boundary IoU from 89.24% to 90.95%, corresponding to an improvement of 1.71 percentage points over the baseline. This result provides empirical evidence that CLF can indirectly benefit boundary-related segmentation, although it is not specifically designed as a boundary loss. When all three components are combined, PSAM achieves the best overall performance (Dice 97.90%), demonstrating strong complementarity among the modules.

**Table 3:** Ablation study evaluating the contribution of each key component to the model's performance under the 1-shot setting.

CGSC	PFEM	CLF	Acc	Prec	Recall	Dice	IoU	Boundary IoU
✗	✗	✗	94.83	94.14	89.94	92.80	90.98	89.24
✓	✗	✗	95.50	95.07	91.82	94.21	92.30	91.56
✗	✓	✗	96.12	96.59	92.31	94.99	93.06	90.12
✗	✗	✓	94.91	94.25	90.14	93.17	91.28	90.95
✓	✓	✗	97.80	97.98	94.89	96.01	93.63	93.20
✓	✗	✓	98.71	98.35	95.38	97.20	94.84	94.25
✗	✓	✓	**99.57**	98.50	96.42	97.75	95.31	93.94
✓	✓	✓	**99.57**	**99.17**	**96.63**	**97.90**	**95.85**	**94.71**

*Note:***The best results are highlighted in bold.**

**Fig. 8:**
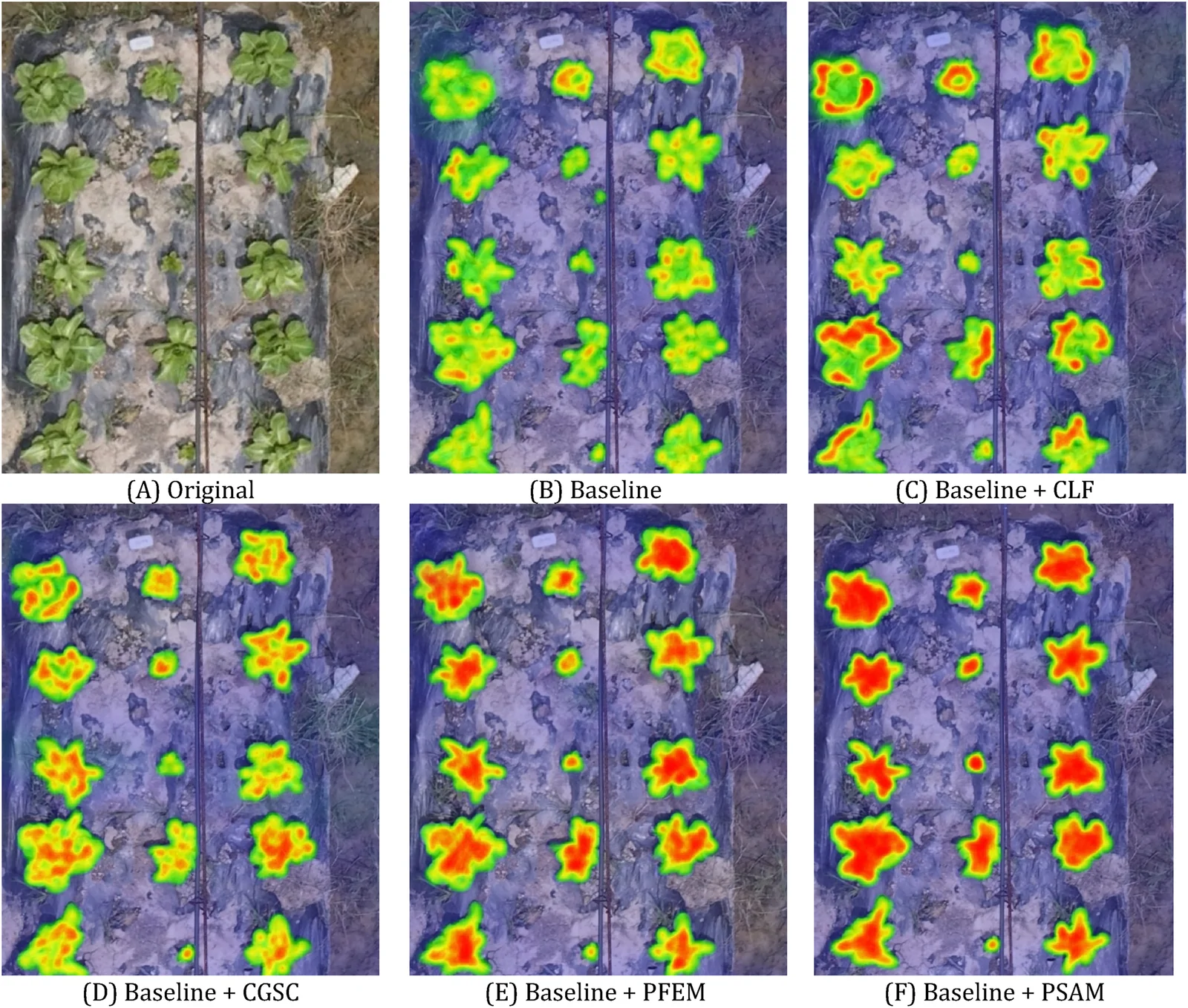
Class activation heatmaps under different module configurations. (A) Original input image. (B) Baseline SAM2 model. (C) Baseline with CLF. (D) Baseline with CGSC module. (E) Baseline with PFEM module. (F) Full PSAM model, showing strong, uniform, and well-localized activations across the plant canopy.

Fig. 8 visualizes class activation heatmaps under different module configurations. The baseline model exhibits weak and dispersed activations (Fig. 8(B)), indicating limited discriminative capability. Incorporating the CLF enhances edge-focused responses (Fig. 8(C)), while the CGSC module strengthens both boundary and interior feature activations (Fig. 8(D)). PFEM provides broader but less refined responses across the target region (Fig. 8(E)). In contrast, the complete PSAM model produces strong, uniform, and well-localized activations over the entire plant (Fig. 8(F)), with clear boundary delineation and effective background suppression, illustrating the synergy of these components.

Overall, the ablation results confirm that the three components play complementary roles: PFEM provides global semantic guidance, CGSC refines local boundary features, and the CLF further improves detail sensitivity. Their integration enables PSAM to achieve robust and accurate segmentation in complex agricultural scenes.

### 3.4 Application-oriented phenotypic analysis enabled by PSAM

Based on the high-precision segmentation results of the PSAM model, this study systematically extracted a total of 15 key phenotypic parameters across three dimensions: morphology, color features, and texture features, as detailed in Table 4. (The specific definitions and calculation formulas for these parameters are provided in Appendix Table A1.) The complete quantitative records for all accessions are provided in Supplementary Table S1 (Excel format). These parameters provide a comprehensive quantitative basis for germplasm screening.

**Table 4:** Definitions and calculation methods for the 15 extracted phenotypic parameters.

Category	Parameter
Morphological	Canopy Area; Compactness; Aspect Ratio; Convexity
Colorimetric	Mean R, G, B; Std. R, G, B; NGRDI; ExG; CIVE
Textural	GLCM Contrast; GLCM Homogeneity

**Fig. 9:**
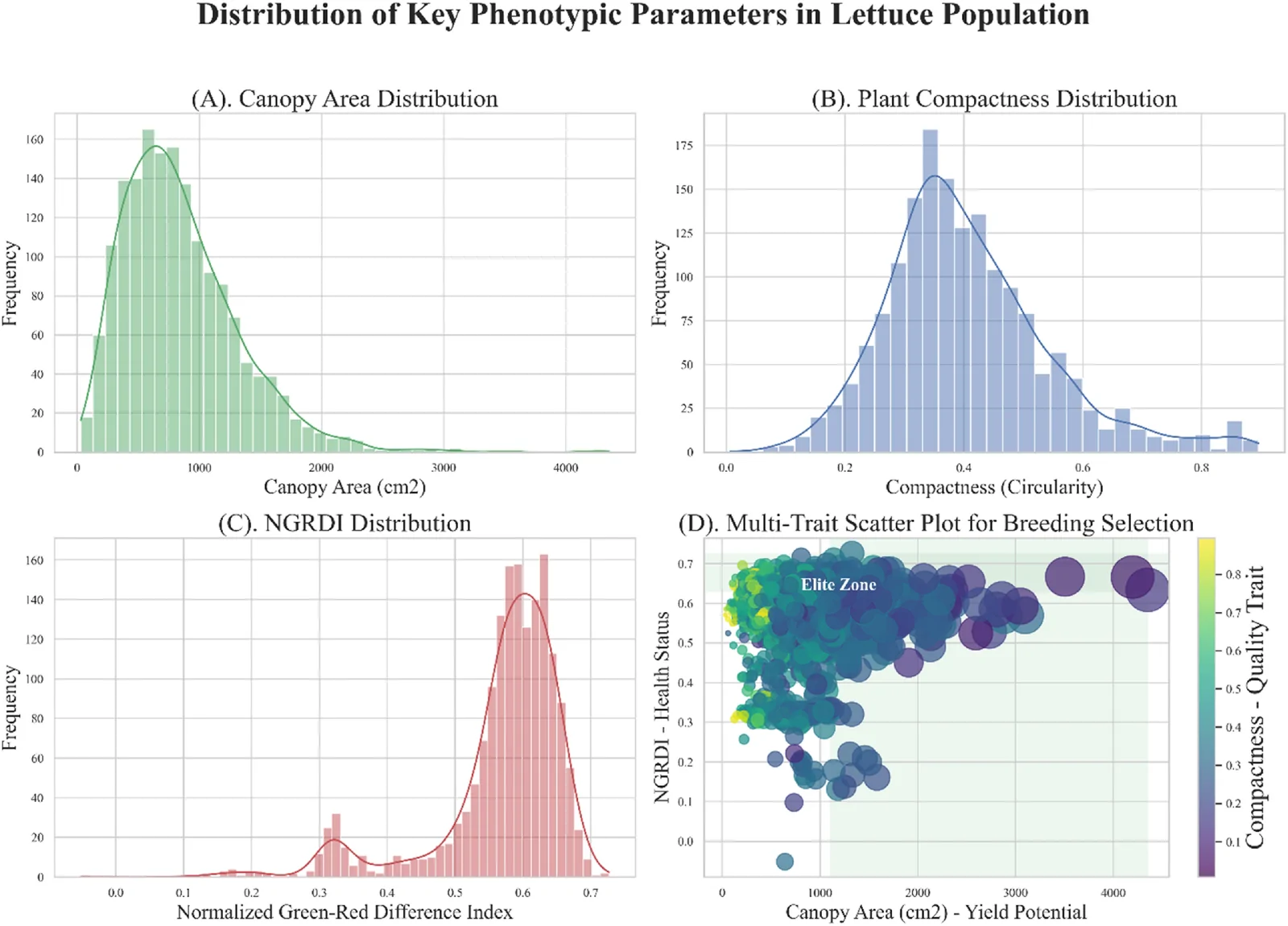
Distribution and multi-trait correlation analysis of key phenotypic parameters extracted from 1884 lettuce plots. (A) Histogram of canopy area. (B) Histogram of plant compactness. (C) Histogram of the Normalized Green-Red Difference Index (NGRDI). (D) Scatter plot illustrating the correlation among canopy area, NGRDI, and compactness. The upper-left region, annotated as the “Elite zone”, highlights germplasm accessions with superior integrated traits for efficient breeding selection.

Fig. 9 illustrates the distributions of three key phenotypic indicators—canopy area, plant compactness, and Normalized Green-Red Difference Index (NGRDI)—along with a multi-trait correlation analysis across 1884 lettuce samples. The results reveal broad phenotypic variation at the evaluated growth stage.

Different germplasm accessions exhibit a continuous distribution in canopy area (Fig. 9(A)), indicating significant variation in growth vigor. The distribution of plant compactness (Fig. 9(B)) is particularly sensitive to head formation, with high values (close to 1.0) corresponding to compact heads, while low values may indicate early bolting. The distribution of NGRDI (Fig. 9(C)) effectively reflects differences in chlorophyll content, providing direct evidence for assessing photosynthetic potential. Through multi-trait joint analysis (Fig. 9(D)), the study further explored the relationships among canopy area, NGRDI, and compactness. This finding highlights the importance of comprehensive multi-trait assessment in breeding. Based on this analysis, researchers can quickly identify germplasm with superior overall traits. Specifically, accessions clustered in the upper-left region of Fig. 9(D) simultaneously exhibit high health status (high NGRDI) and compact head formation (high compactness) without the excessive canopy expansion associated with early bolting. Pinpointing this “Elite zone” significantly improves germplasm selection efficiency. These results underscore the necessity of integrated multi-trait evaluation in breeding and demonstrate that the PSAM-based high-throughput phenotyping system provides a solid data foundation and technical support for exploring complex agronomic trait patterns.

## 4 Discussion

### 4.1 Segmentation performance on different lettuce varieties

**Fig. 10:**
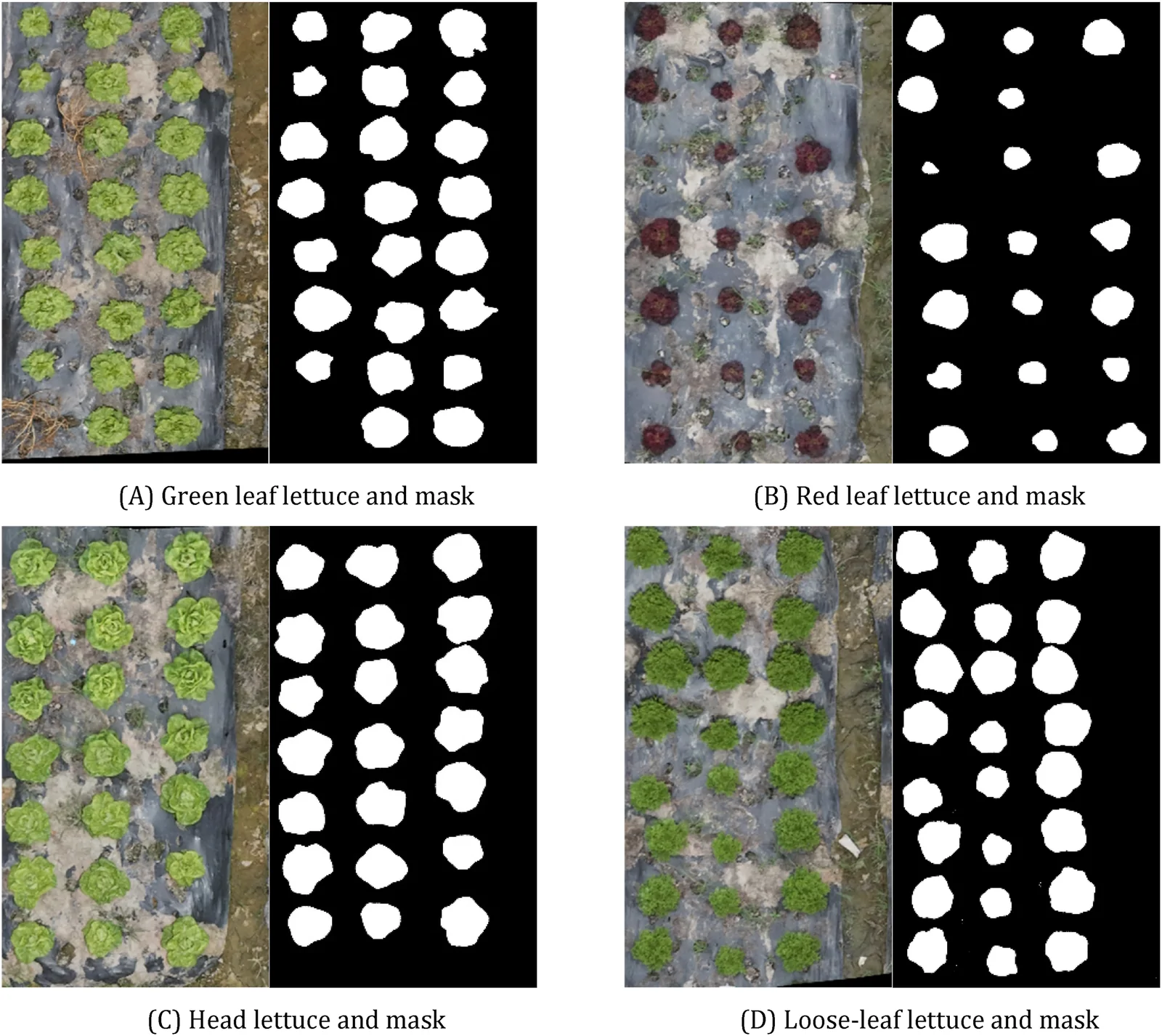
Segmentation results of four representative lettuce varieties with diverse phenotypic traits. For each variety, the left panel shows the original plot image, and the right panel shows the segmentation mask predicted by PSAM. (A) Green leaf lettuce. (B) Red leaf lettuce. (C) Head lettuce. (D) Loose-leaf lettuce.

To evaluate PSAM's generalization ability across lettuce varieties with diverse phenotypic traits, four representative types were selected for testing, covering green leaf lettuce, red leaf lettuce, head lettuce, and loose-leaf lettuce. The segmentation visualization results are shown in Fig. 10.

Results indicate that PSAM maintains stable performance across all varieties. For green and red leaf lettuce, as shown in Fig. 10(A) and (B), the model effectively overcomes color interference and accurately captures leaf edge details. For head lettuce, it precisely identifies the compact spherical shape, avoiding under-segmentation, as shown in Fig. 10(C). For loose-leaf lettuce, it effectively restores the irregular canopy structure with minimal local omissions, as shown in Fig. 10(D).

These results validate the effectiveness of the core mechanism of the PFEM: By retrieving and integrating instance-level visual priors from an external feature library, the model can infer target characteristics from similar prototype priors, thereby improving segmentation performance for varieties with significant phenotypic differences. This approach overcomes the limitation of traditional few-shot models, which rely solely on limited labeled samples to learn all features. Meanwhile, the CGSC module, with its strong detail perception capability, ensures accurate delineation of high-order features such as leaf boundaries across different morphological structures. The synergistic working mechanism of these two modules allows the model to maintain stable segmentation performance across phenotypically diverse lettuce varieties, providing key technical support for high-throughput phenotypic analysis of large-scale, multi-variety germplasm resources.

### 4.2 Comparative analysis with state-of-the-art methods in complex scenarios

Although recent few-shot semantic segmentation (FSS) models have made significant progress, their direct application in heterogeneous agricultural environments often yields suboptimal results. Because complex environmental factors—such as weeds, shadows, and overlapping leaves—often co-exist in real-world agricultural imagery, it is challenging to rigidly decouple the dataset into isolated single-condition subsets. Accordingly, the methods were qualitatively compared under four representative and often co-occurring conditions—weed interference, shadows, overlapping leaves, and blurred boundaries—using the enlarged results in Fig. 7, while the overall Boundary IoU results in Table 2 provided quantitative support.

Traditional CNN-based FSS networks, such as PANet and CANet, rely heavily on masked average pooling to extract a single deterministic prototype. As demonstrated in Table 2, these methods achieve Dice scores of only 81.21% and 84.17% under the 1-shot setting, respectively. This performance degradation occurs because a single prototype cannot encompass the high intra-class variance of lettuce cultivars, especially when dealing with dense overlapping leaves and drastic morphological differences between heading and loose-leaf types.

Diffusion-based models like DiffewS generate better visual representations and achieve a high Dice score of 96.34%. However, as illustrated in the complex marginal regions (Fig. 7), DiffewS still struggles with blurred boundaries in densely planted plots. Furthermore, under extremely complex visual conditions—such as severe weed interference and intense shadowing caused by adjacent leaves—baseline general foundation models (e.g., SAM2) often suffer from “green-on-green” misclassifications and background noise blending.

In contrast, the proposed PSAM systematically addresses these bottlenecks across all aforementioned adverse conditions. By integrating the external Prototype Feature-Enhanced Prompting Module (PFEM) with SAM2, PSAM dynamically retrieves instance-level priors, maintaining high target sensitivity against weed interference. Moreover, PSAM overcomes shadow and occlusion issues through the Complex Gated Spatial-Channel Attention Module (CGSC), which dynamically downweights shadow-affected channels and amplifies spatial edge responses via variance maps, thereby sharply delineating overlapping boundaries. This superior edge-preserving capability in complex scenarios is quantitatively supported by the 94.71% Boundary IoU (outperforming SAM2 by 5.47%, Table 2), confirming that integrating task-specific visual retrieval with boundary-aware attention mechanisms is highly effective for complex agricultural applications.

Compared with recent agricultural segmentation approaches, including DeepLabV3+-based lettuce segmentation [27], the Agricultural Segment Anything Model Adapter [33], and YOMASK [37], PSAM specifically focuses on few-shot segmentation of complex open-field UAV imagery; however, direct numerical comparisons are inappropriate because these studies used different datasets and evaluation protocols.

### 4.3 Limitations

Despite the excellent performance of the PSAM model in lettuce segmentation tasks, this study has several limitations. First, model validation was primarily conducted on a single crop (lettuce), and its generalization ability across crops with different morphological features (e.g., wheat, maize) or more complex scenarios (e.g., diseased or pest-affected leaves) remains to be further verified. Second, the current feature library is constructed passively based on accumulated training data, lacking an active selection mechanism for critical samples, which may affect the density and retrieval efficiency of the feature library. In addition, the interaction between the prototype feature enhancement and attention modules remains relatively shallow, and deeper feature synergistic optimization has not yet been achieved.

To address these limitations, future work will be carried out in three directions:

(1) extending the framework to multiple typical crops to systematically evaluate cross-species adaptability;
(2) introducing active learning strategies, such as uncertainty-based sampling, to optimize feature library construction and improve the quality and efficiency of prior knowledge;
(3) exploring deeper coupling between prototype features and attention mechanisms at the model structure level, designing more interactive feature enhancement pathways.

These improvements are expected to further enhance the practical value and applicability of the model in smart agriculture.

## 5 Conclusion

This study addresses key challenges in agricultural few-shot segmentation, including limited domain-specific knowledge, insufficient boundary perception, and high phenotypic heterogeneity across crop varieties, by proposing a prototype-enhanced segmentation framework termed PSAM. Built upon the SAM2 architecture, PSAM incorporates three complementary components that jointly improve segmentation robustness under complex field conditions. First, PFEM incorporates instance-level visual priors from an external feature library, effectively compensating for the scarcity of agricultural domain knowledge in the base model. Second, the CGSC module is designed to dynamically integrate spatial and channel-wise information, enhancing sensitivity to diverse lettuce boundaries and fine structural details. Third, CLF jointly optimizes pixel-level classification and region-level overlap, improving robustness to challenging samples such as leaf edges. Experimental results on a large-scale field lettuce dataset demonstrate that PSAM achieves a Dice coefficient of 97.90% under the 1-shot setting, substantially outperforming the SAM2 baseline. This performance advantage is consistently observed across phenotypically diverse lettuce varieties, demonstrating stable segmentation performance under heterogeneous breeding-field conditions. Furthermore, the high-precision segmentation results enable the automated extraction of 15 phenotypic parameters, providing a reliable data foundation for breeding-oriented phenotypic analysis in lettuce. Future work will extend this framework to additional crop species and investigate deeper interactions between prototype representations and internal model computations, with the aim of further enhancing the practicality and scalability of the proposed approach in smart agriculture.

## Author contributions

T.X.: Conceptualization, Methodology, Formal analysis, Visualization, Writing-original draft. J.Q.: Methodology, Investigation, Data curation, Software, Visualization, Validation, Writing - review and editing. S.B.: Methodology, Data curation, Investigation, Resources, Writing – review and editing. M.Y.: Formal analysis, Writing – review and editing. L.L.: Resources, Funding acquisition. D.H.: Software, Validation. J. N.: Investigation, Software, Validation. G.H.: Validation, Resources. H.Y.: Investigation, Software. S.H.: Resources. G.G.: Resources. S.W.: Conceptualization, Resources, Funding acquisition. M.T.: Conceptualization, Methodology, Investigation, Visualization, Project administration, Resources, Supervision, Writing – review and editing.

## Data availability statement

The test dataset used in this study is freely available at Zenodo via https://doi.org/10.5281/zenodo.18371786. The trained model weights are available at https://doi.org/10.5281/zenodo.18371750. We provide open access to these resources to foster collaboration, replication, validation, and further exploration of our findings. The raw image data supporting the conclusions of this article will be made available by the authors, without undue reservation. The extracted phenotypic dataset is available in the Supplementary Material of this article.

## Funding

This research was funded by the Shanghai Science and Technology Support Project [grant number 25N22800400], and the Shanghai Academy of Agricultural Sciences Program for Excellent Research Team [grant number 2022015].

## Declaration of competing interest

The authors declare that they have no known competing financial interests or personal relationships that could have appeared to influence the work reported in this paper.

## Figures and Tables

**Table A1:** Definitions and calculation formulas for the 15 extracted phenotypic parameters.

Category	Parameter	Definition	Formula/Method
Morphological	Canopy Area	The total physical area of the plant canopy.	$A=N_{pixels}\times {GSD}^{2}$
	Compactness	A measure of how circular the plant shape is, indicating heading tightness. Values are close to 1 for perfect circles.	$C=\frac{4\pi A^{P}}{P^{2}}$
	Aspect Ratio	The ratio of the bounding box width to its height, describing the overall plant shape.	$AR=\frac{{Width}_{bbox}}{{Height}_{bbox}}$
	Convexity	The ratio of the mask area to its convex hull area, quantifying the solidity of the canopy shape.	$Convexity=\frac{A_{p}}{A_{hull}}$
Colorimetric	Mean R, G, B	The average intensity values of the Red, Green, and Blue channels within the canopy mask.	$\bar{C}=\frac{1}{N_{pixels}}\sum_{i=1}^{N_{pixels}}I_{c}\left(i\right)$
	Std. R, G, B	The standard deviation of the R, G, B channels, measuring the color uniformity of the canopy.	$\sigma_{c}=\sqrt{\frac{1}{N_{pixels}}\sum_{i=1}^{N_{pixels}}{\left(I_{c}\left(i\right)-\bar{C}\right)}^{2}}$
	NGRDI	Normalized Green-Red Difference Index, strongly correlated with chlorophyll content and plant vigor.	$NGRDI=\frac{G-R}{G+R}$
	ExG	Excess Green Index, another vegetation index robust for separating plants from soil background.	$ExG=\frac{2G-R-B}{R+G+B}$
	CIVE	Color Index of Vegetation Extraction, designed to be robust to variations in lighting and shadows.	CIVE=0.441R−0.811G+0.385B+18.787
Textural	GLCM Contrast	Measures the local intensity variations in the grayscale canopy image, reflecting surface roughness.	${GLCM}_{Contrast}=\sum_{i,j}{\left(i-j\right)}^{2}p\left(i,j\right)$
	GLCM Homogeneity	Measures the closeness of the distribution of elements in the GLCM to the GLCM diagonal.	${GLCM}_{Homogeneity}=\frac{p\left(i-j\right)}{1+|i-j|}$

## Appendix B Supplementary data

Table S1 Complete quantitative records for all accessions.
